# The multifaceted role of intracellular glycosylation in cytoprotection and heart disease

**DOI:** 10.1016/j.jbc.2024.107296

**Published:** 2024-04-18

**Authors:** Priya Umapathi, Akanksha Aggarwal, Fiddia Zahra, Bhargavi Narayanan, Natasha E. Zachara

**Affiliations:** 1 Division of Cardiology, Department of Medicine, The Johns Hopkins University School of Medicine, Baltimore, Maryland, USA; 2 Department of Biological Chemistry, The Johns Hopkins University School of Medicine, Baltimore, Maryland, USA; 3 Department of Oncology, The Johns Hopkins University School of Medicine, Baltimore, Maryland, USA

**Keywords:** glycoprotein, ER stress, integrated stress response, heart failure, hypertrophy, cardioprotection, cellular stress response, chaperone, autophagy

## Abstract

The modification of nuclear, cytoplasmic, and mitochondrial proteins by O-linked β-N-actylglucosamine (O-GlcNAc) is an essential posttranslational modification that is common in metozoans. O-GlcNAc is cycled on and off proteins in response to environmental and physiological stimuli impacting protein function, which, in turn, tunes pathways that include transcription, translation, proteostasis, signal transduction, and metabolism. One class of stimulus that induces rapid and dynamic changes to O-GlcNAc is cellular injury, resulting from environmental stress (for instance, heat shock), hypoxia/reoxygenation injury, ischemia reperfusion injury (heart attack, stroke, trauma hemorrhage), and sepsis. Acute elevation of O-GlcNAc before or after injury reduces apoptosis and necrosis, suggesting that injury-induced changes in O-GlcNAcylation regulate cell fate decisions. However, prolonged elevation or reduction in O-GlcNAc leads to a maladaptive response and is associated with pathologies such as hypertrophy and heart failure. In this review, we discuss the impact of O-GlcNAc in both acute and prolonged models of injury with a focus on the heart and biological mechanisms that underpin cell survival.

Perturbations in cellular homeostasis that result from environmental and physiological injury lead to complex, system-wide, remodeling of cellular pathways that assess and counteract damage and, if necessary, remove terminally damaged cells *via* programmed cell death (1, 2). This process, known as the cellular stress response, is key to maintain cellular homeostasis and plays a critical role in the pathophysiology of disease (1, 2). Underscoring the latter, key regulators of the stress response are being targeted for the treatment of common pathologies that include cancer, neurodegenerative disease(s), diabetes, autoimmune disorders, ischemic damage, and heart failure (3, 4, 5, 6).

Macromolecular damage results in cell cycle arrest, repair and/or stabilization of damaged macromolecules, and redirection of energy metabolism to support repair (2). Affecting these changes are protein–protein interactions, protein localization, protein turnover, and posttranslational modifications (PTMs) that sense and signal damage, and subsequently enact repair or cell death (2, 7). While the impact of PTMs such as phosphorylation is/are well established regulators of the cellular stress response (7, 8), the role of glycosylation has been less well studied. Changes in cell surface glycans play roles as damage-associated molecular patterns and in the recruitment of inflammatory cells after injury (9, 10). Within the cell, release of glycoconjugates from lysosomes act as a damage-associated molecular pattern (11); however, it is the modification of nuclear, cytoplasmic, and mitochondrial proteins by O-linked β-N-acetylglucosamine (O-GlcNAc) that has emerged as critical target of the cellular stress response (12, 13).

The O-GlcNAc-modification, or O-GlcNAcylation, increases in a dose- and time-dependent manner in response to myriad environmental stressors (12). Acute augmentation of O-GlcNAc using genetic or pharmacological approaches reduces cell death (Fig. 1), whereas reducing O-GlcNAcylation sensitizes cells to injury. These observations have been made in a broad range of mammalian cell types (primary and transformed), as well as physiological models of injury, suggesting that stress-induced O-GlcNAc cycling is a conserved response of mammalian cells/tissues (13, 14). Stress-induced changes in O-GlcNAcylation impact pathways such as transcription, translation, signal-transduction, proteostasis, and metabolism (13, 14). Collectively, these data support the notion that O-GlcNAc is used by cells to rewire cellular pathways in response to injury—a process by which we term the O-GlcNAc–mediated stress response. In this review, we highlight key proteins and pathways regulated by O-GlcNAc during injury that impact the cellular response to injury and discuss O-GlcNAc-cycling in the context of acute myocardial injury and the pathophysiology of heart disease.

**Figure 1:**
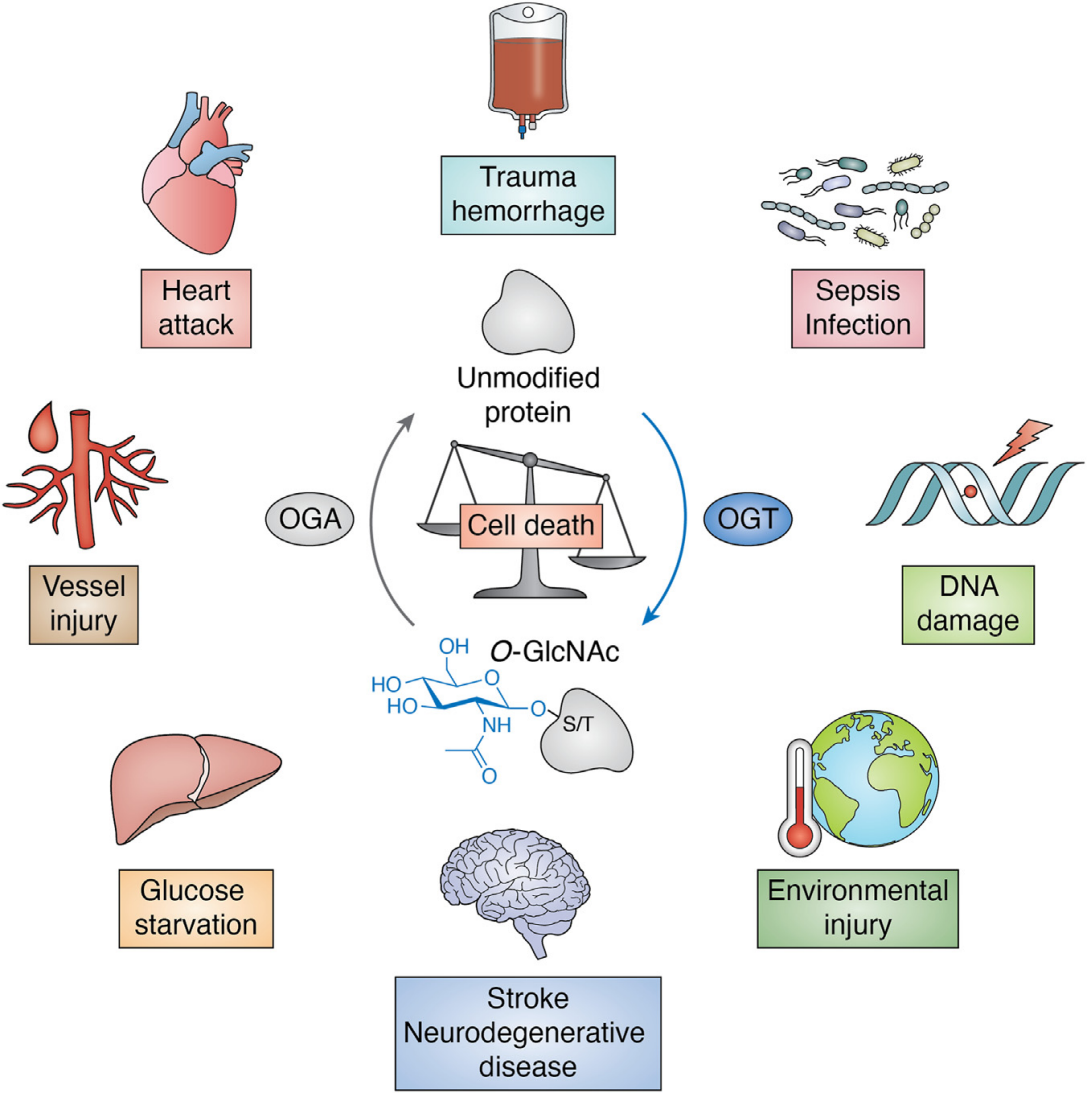
**Cellular stress/injury models impacted by O-GlcNAcylation.** Stress-induced changes in O-GlcNAc-cycling and O-GlcNAc–mediated cytoprotection have been observed in a broad range of environmental and physiological models. While global changes in O-GlcNAc levels are detected (both increased and decreased) in response to injury, protein specific studies identify a more nuanced model in which O-GlcNAc is cycled on and off proteins differentially. That is, while global levels of O-GlcNAc are increased, O-GlcNAcylation can be reduced on a subset of proteins. Similarly, in models where global O-GlcNAc levels are decreased, an elevation of O-GlcNAcylation is observed on a subset of proteins. Nonetheless, in all these models manipulating O-GlcNAc impacts survival, specifically acutely depressing O-GlcNAc promotes cell death, whereas acutely increasing O-GlcNAc augments survival. One interpretation of these data is that loss of O-GlcNAc on a subset of proteins promotes cell death, whereas elevation of O-GlcNAc on others promotes cell survival pathways. OGA, O-GlcNAcase; OGT, O-GlcNAc-transferase.

### The impact of injury on O-GlcNAc cycling

Rapid and dynamic changes to O-GlcNAc, in response to environmental stress, suggests that stress-induced O-GlcNAcylation results from changes in the abundance or regulation of the enzymes that control O-GlcNAc cycling (12, 15). Below, we introduce the O-GlcNAc transferase (OGT; EC:2.4.1.255) (16, 17, 18) and O-GlcNAcase (OGA; EC:3.2.1.169) (19, 20, 21), the enzymes that catalyze the addition and removal of O-GlcNAc, respectively (Fig. 2). We discuss the impact of cellular injury on OGT and OGA, as well as the synthesis of the nucleotide sugar used by OGT: UDP-GlcNAc (16, 17, 18).

**Figure 2:**
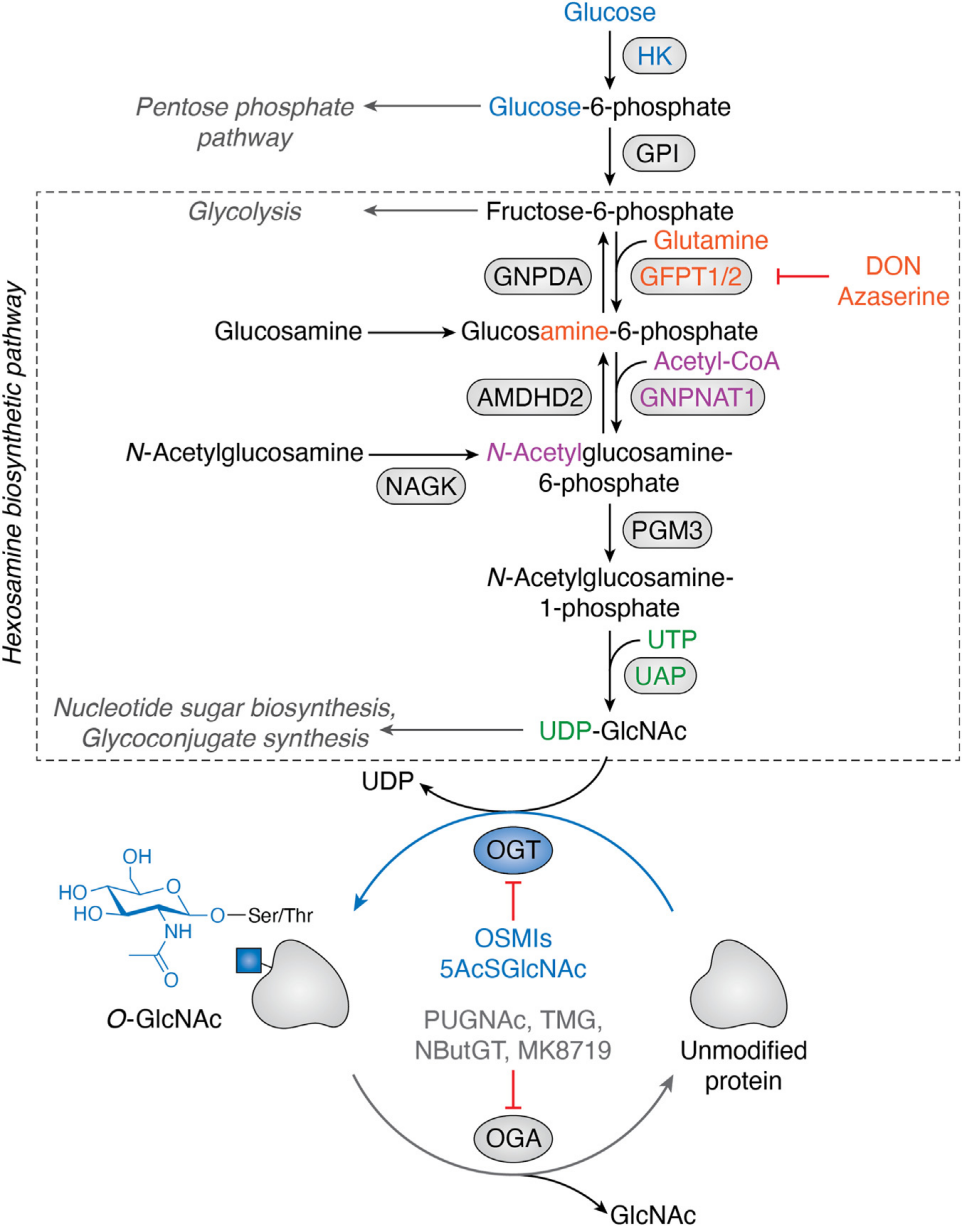
**The biosynthesis of O-GlcNAc.** O-GlcNAc transferase (OGT; O15294, EC:2.4.1.255) and O-GlcNAcase (OGA; O60502; EC:3.2.1.169), respectively, catalyze the addition and removal of O-GlcNAc. OGT uses the nucleotide sugar UDP-GlcNAc, which is synthesized by the hexosamine biosynthetic pathway (HBP, *boxed*). Select OGT and OGA inhibitors are indicated, as well as inhibitors of glutamine-fructose-6-phosphate aminotransferase (*GFPT1/2*; Q06210/O94808, EC:2.6.1.16), the rate limiting enzyme of the HBP. Other enzymes include: hexokinase (*HK*; EC:2.7.1.1), glucose-6-phosphate isomerase (*GPI*; EC:5.3.1.9), glucosamine-6-phosphate isomerase (*GNPDA1/2*; P46926/Q8TDQ7, EC:3.5.99.6), N-acetyl-D-glucosamine kinase (*NAGK*; Q9UJ70, EC:2.7.1.59), glucosamine 6-phosphate N-acetyltransferase (*GNPAT*; Q96EK6, EC:2.3.1.4), N-acetylglucosamine-6-phosphate deacetylase (*AMDHD2*; Q9Y303, EC:3.5.1.25), phosphoacetylglucosamine mutase (*PGM3*; O95394; EC:5.4.2.3), UDP-N-acetylhexosamine pyrophosphorylase (*UAP*; Q16222; EC:2.7.7.83). UniProt identification numbers are listed for human proteins/genes. 5AcSGlcNAc, (2S,3R,4R,5S,6R)-3-acetamido-6-(acetoxymethyl)tetrahydro-2H-thiopyran-2,4,5-triyl triacetate; AZA, azaserine; DON, 6-diazo-5-oxo-L-norleucine; NButGT, (3aR,5R,6S,7R,7aR)-3a,6,7,7a-tetrahydro-5-(hydroxymethyl)-2-propyl-5H-pyrano[3,2-d]thiazole-6,7-diol; OMSI: (αR)-α-[[(1,2-dihydro-2-oxo-6-quinolinyl)sulfonyl]amino]-N-(2-furanylmethyl)-2-methoxy-N-(2-thienylmethyl)-benzeneacetamide; PUGNAc, O-(2-acetamido-2-deoxy-D-glucopyranosylidenamino) N-phenylcarbamate; TMG, thiamet-G.

#### OGT, the writer

OGT is a highly conserved enzyme vital for mammalian development that resides on the X chromosome (22). Beyond its role as a glycosyltransferase, OGT acts as a scaffolding protein and uses UDP-GlcNAc to proteolytically cleave host cell factor 1 (23, 24, 25, 26). OGT forms a homodimer (27); each subunit is comprised of an N-terminal tetratricopeptide repeat (TPR) domain, a linker region, and a C-terminal catalytic domain (17, 18, 28). OGT has three well-characterized isoforms each differing in the size of their TPR domain: the nuclear and cytoplasmic isoform (1046 aa or 1034 aa), the short isoform (665 aa), and the mitochondrial isoform (900 aa) (29, 30). Despite the lack of a well-defined consensus sequence for O-GlcNAcylation, OGT can differentially interact with and modify many proteins (31, 32, 33). OGT can engage with its substrate targets through multisite interactions in three distinct modes: extensive interaction with the TPR domain, moderate interaction with the TPR domain, or direct interaction with the catalytic domain (34, 35, 36). Underscoring the importance of TPR interactions, mutations within this domain are associated with X-linked intellectual disability (37).

The regulation of OGT function centers around four key themes. Firstly, OGT abundance, which can be tuned at both the transcriptional and translational level. For example, reduced O-GlcNAc levels promote the maturation of *OGT* mRNA through the splicing of detained introns within *OGT* transcripts, ultimately resulting in an increase in OGT protein expression (38, 39, 40). Secondly, OGT is directly modified and regulated by PTMs. For example, phosphorylation of OGT by glycogen synthase kinase3β (GSK3β) leads to increased OGT activity (41). Thirdly, changes in OGT's subcellular localization likely results in the O-GlcNAcylation of different substrate complements. For instance, OGT has been demonstrated to localize to the plasma membrane upon insulin signaling (42). Lastly, protein interactors of OGT are known to target OGT to different substrates. Specifically, during periods of nutrient deprivation, OGT interacts with p38 mitogen-activated protein kinase (p38 MAP kinase), which subsequently influences the selection of OGT substrates such as neurofilament H (43).

Regulating OGT during injury or stress is a complex, context-dependent process. Acute conditions like oxidative stress, ischemic injury, nutrient deprivation, and heat stress can impact OGT regulation differently. For instance, cellular thermal stress models suggest that OGT abundance remains stable while its activity increases in response to heat stress (12). On the other hand, under conditions of oxidative stress in cell line models, both OGT activity and abundance show a time-dependent increase concomitant with changes in the protein interactome (12, 33, 44). During myocardial ischemia, OGT activity is depressed concomitant with a reduction in O-GlcNAcylation (45). Nutrient deprivation appears to predominantly target OGT abundance, with increased O-GlcNAcylation associated with an increase in *OGT* mRNA and OGT protein abundance (43). Similarly, chronic conditions such as hypertension, aortic stenosis, and aortic constriction exhibit increased OGT expression (46). Conversely, ischemic preconditioning (IPC) models demonstrate elevated OGT expression and activity, followed by increased O-GlcNAc levels, which are associated with cardioprotection as evident by a decrease in infarct size after ischemia reperfusion (I/R) injury (47, 48). One interpretation of these data is that the regulatory pathways controlling OGT are tissue- or stress-dependent. Another is that acute stressors are more likely to impact OGT activity and substrate targeting, whereas chronic stressors impact OGT abundance.

#### OGA, the eraser

OGA is a soluble N-acetylglucosaminidase with a neutral pH optimum (19, 20, 21). There are two well-characterized isoforms of OGA: full-length OGA (916 aa) and short OGA (677 aa) (19, 20, 49). While short OGA preferentially localizes to the nucleus and lipid droplets (49), the predominant OGA isoform (916 aa) is found throughout the cell but is enriched in the cytosol (19, 20, 50). Full-length OGA is comprised of two distinct domains: an N-terminal catalytic domain with homology to hyaluronidases (51) and a C-terminal domain with homology to histone acetyltransferases (52). The intervening sequence comprises of a region of low complexity, followed by a stalk domain, which is critical for dimerization (53) and substrate selectivity (54). While OGA is cleaved by caspase 3 during apoptosis, the physiological significance of these observations is unknown (20, 55).

In cellular models of oxidative stress, counterintuitively, OGA activity and abundance are modestly increased (33, 44). While global levels of O-GlcNAc are enhanced in these models, proteomic studies demonstrate that O-GlcNAcylation on a subset of proteins decreases (56). These data suggest that OGA is targeted to specific proteins during times of stress. Consistent with this hypothesis the OGA protein interactome is extensively remodeled in response to oxidative stress. Validation of the OGA interactome demonstrated that fatty acid synthase, a stress-induced protein interactor of OGA, inhibited OGA hexosaminidase activity and glycosylation of a subset of proteins (44). In physiological models such as I/R injury, IPC, and remote IPC (rIPC, IPC of noncardiac tissue that promotes cardioprotection) that lead to elevated levels of O-GlcNAc, no change in OGA abundance has been reported (45, 47, 48). However, a cell culture model of I/R injury and a murine infarct–induced heart failure displayed enhanced O-GlcNAcylation. In this model, microRNA (miRNA)-539 levels are upregulated resulting in a depression in OGA protein abundance, contributing to elevated O-GlcNAcylation (57). OGA activity does not appear to be targeted in IPC but is depressed in rIPC (47, 48). Collectively, these data suggest cells and tissues target OGA abundance, activity, and substrate targeting to affect stress-induced changes in O-GlcNAcylation; however, further work is required to understand the signaling pathways and biological mechanisms that coordinate such changes.

#### HBP, the source of UDP-GlcNAc

The hexosamine biosynthetic pathway (HBP) is a branch of glycolysis that synthesizes UDP-GlcNAc. The first and rate limiting step of the HBP is the conversion of fructose-6-phosphate to glucosamine-6-phosphate, a reaction catalyzed by glutamine fructose-6-phosphate amidotransferase (GFAT/*GFPT*) (58). Glucosamine-6-phosphate is acetylated by glucosamine-6-phosphate N-acetyltransferase (GNPNAT) to generate N-acetylglucosamine-6-phosphate. N-Acetylglucosamine-6-phosphate undergoes deacetylation to produce N-acetylglucosamine-1-phosphate, a step catalyzed by phosphoglucomutase 3 (PGM3). The final step of the pathway is catalyzed by UDP-N-acetylglucosamine pyrophosphorylase (UAP/AGX) and produces UDP-GlcNAc (59). At two points, flux through the HBP can be reversed: Firstly, N-acetylglucosamine-6-phosphate deacetylase (amidohydrolase domain–containing 2; AMDHD2) deacetylates N-acetylglucosamine-6-phosphate to generate glucosamine-6-phosphate (60); Secondly, glucosamine-6-phosphate deaminase (GNPDA) catalyzes the conversion of glucosamine-6-phosphate to fructose-6-phosphate and ammonia (61). Critically, glycans from glycoprotein breakdown can enter the HBP by phosphorylation of glucosamine and GlcNAc (62, 63). Conveniently, these salvage pathways can be used to supplement UDP-GlcNAc levels and to introduce unnatural sugars that facilitate click chemistry (Fig. 2) (64).

We are only just beginning to understand the sophisticated signaling network that controls the HBP, allowing cells and tissues to fine-tune nucleotides sugar levels based on their needs. GFAT has two isoforms, GFAT1 and GFAT2. While GFAT1 is ubiquitous, GFAT2 is highly expressed in the central nervous system. Critical to the discussion below, GFAT2 plays a key role in regulating O-GlcNAcylation in the heart, with elevated expression of GFAT2 (not GFAT1) observed in response to cardiac hypertrophy (65). Importantly, UDP-GlcNAc feedback inhibits GFAT1 (58) and to a lesser extent GFAT2 (66). GFAT is composed of a glutaminase domain and an isomerase/transferase domain. The orientation of these domains controls the rate of catalysis and is impacted by both substrate binding and PTM. Binding of fructose-6-phosphate to GFAT1 results in a conformational shift bringing the two domains together, thus promoting catalysis. UDP-GlcNAc prevents this conformational shift by binding the isomerase/transferase domain. Mutation of GFAT1 (R203H) alleviates feedback inhibition by UDP-GlcNAc (67). GFAT2 displays a reduced catalytic rate and is less sensitive to UDP-GlcNAc. The latter has been attributed to reduced flexibility of a key regulatory loop that is further stabilized by interaction with Arg342. As GFAT2 is less sensitive to feedback inhibition by UDP-GlcNAc, it has been proposed that cells with high GFAT2 abundance are more reliant on amidohydrolase domain–containing 2 to regulate HBP flux (67).

GFAT is also regulated by protein phosphorylation. In *Caenorhabditis elegans,* PKA phosphorylates GFAT1 at residue Ser205 to prevent UDP-GlcNAc feedback inhibition and increase enzymatic activity during proteotoxic stress (67). During glucose starvation and glycolysis inhibition, 5′ AMP-dependent protein kinase (AMPK) is activated and phosphorylates GFAT1. Phosphorylation of GFAT decreases GFAT1 activity, presumably to ensure that the limited amount of available glucose is metabolized *via* glycolysis rather than the HBP (68). In contrast, during glutamine deprivation the mammalian target of rapamycin complex (mTORC) 2 phosphorylates GFAT1, leading to activation and enhanced HBP flux (69). In addition to PTMs, several forms of stress impact the abundance of GFAT1. Mice exposed to low oxygen (hypoxia) for 4 weeks have increased *GFAT1* mRNA and O-GlcNAcylation. The latter exacerbates right ventricular dysfunction and remodeling *via* enhancement of hypertrophy, mitophagy, and fibrosis (70). Many cancer cells have elevated levels of enzymes in the HBP, potentially to combat common features of tumors: proteotoxicity, hypoxia, and oxidative stress (71, 72, 73).

To date, most data demonstrating changes in HBP flux associated with cellular injury arise from studies on endoplasmic reticulum (ER) stress and the unfolded protein response (UPR). For instance, in *Saccharomyces cerevisiae,* the induction of ER stress with tunicamycin leads to upregulation of Slt2, a mitogen-activated protein kinase, which activates GFAT1 and increases flux through the HBP (74). ER stress also targets the abundance of proteins within the HBP. For example, in primary monocytes exposed to the glycolytic inhibitor 2-deoxy-d-glucose, *GFPT1*, *GNPNAT1*, and *PGM3* mRNA levels are elevated (75). In a model of I/R injury, during reperfusion the UPR induces robust splicing of the X-box binding protein 1 (XBP1), resulting in the mature spliced form. In turn, XBP1s acts as a transcription factor to induce genes that include *GFAT1, GNPNAT1, PGM3*, and UDP-galactose-4 epimerase to activate the HBP and increase O-GlcNAcylation (76). In another model of I/R injury, transcript induced in spermiogenesis 40 (Tisp40), an ER-localized transcription factor, displays increased expression, cleavage, and nuclear accumulation. Transcript induced in spermiogenesis 40 binds to a conserved domain of the UPR element of the GFAT1 promoter to increase *GFAT1* expression. In turn, this is thought to increase flux through HBP pathway and O-GlcNAcylation of cardiac proteins, which is thought to protect against oxidative stress and apoptosis during injury (77). Collectively, these data suggest that cells upregulate HBP flux by targeting the activity and abundance of key enzymes.

### Cytoprotection

Studies focused on identifying proteins differentially O-GlcNAcylated in models of heat stress, oxidative stress, trauma hemorrhage, and sepsis demonstrate that diverse proteins are targeted by O-GlcNAc in response to injury (56, 78, 79, 80). These studies have also determined that stress-induced remodeling of the O-GlcNAcome is more nuanced than originally thought. Immunoblotting reveals both global elevation (heat shock, I/R injury, IPC, rIPC) and depression (ischemia/sepsis/trauma hemorrhage) of O-GlcNAcylation in response to injury (Fig. 1); however, protein specific studies demonstrate that O-GlcNAc is cycled on and off proteins differentially. That is, the initiation, duration, amplitude, and magnitude of O-GlcNAcylation are protein, stress, and tissue-dependent (56, 78, 79, 80). As elevating O-GlcNAc promotes cell survival (13, 14, 81), one interpretation of these data is that loss of O-GlcNAc on a subset of proteins promotes cell death, whereas elevation of O-GlcNAc on others promotes cell survival pathways. Indeed, in models of cardiac ischemia, where a decrease in O-GlcNAc is observed late in ischemia (30 min) (82), augmenting O-GlcNAc before or after injury reduces cell death and improves heart function (82, 83, 84, 85, 86). Below, we discuss mechanistic studies that describe the impact of stress-induced O-GlcNAc cycling on protein function, which in turn regulates key pathways in the cellular stress response. We focus on the role of O-GlcNAc in regulating the abundance and activity of chaperones, protein aggregation, and autophagy. The impact of O-GlcNAc on metabolism in cancer cells, inflammasome formation, and mitochondrial function has been reviewed recently (13).

#### Molecular chaperones

##### Chaperone abundance

Initial studies describing the impact of heat shock on O-GlcNAcylation reported that the induction of heat shock protein (HSP) 70 and HSP40 were impacted by the levels of O-GlcNAc (12). In both fibroblasts and hearts, higher O-GlcNAcylation correlated with increased abundance of molecular chaperones such as HSP70 (12, 83, 87). Several mechanisms that underpin these observations have been identified. The injury-induced transcription of molecular chaperones, such as HSP70, requires the transcription factor heat shock factor 1 (HSF1). Activation of HSF1 is multifaceted with key steps including trimerization, nuclear localization, and PTM (7). While evidence for O-GlcNAcylation of HSF1 is indirect (88), deletion of *OGT* in mouse embryonic fibroblasts results in reduced transcript levels of *HSPA1A* (HSP70) and 17 other chaperones (87). Nuclear translocation of HSF1 is not impacted by deletion of OGT; however, inhibitory phosphorylation at Ser303 by GSK3β is reduced in OGT null cells. These data suggest that O-GlcNAcylation suppresses GSK3β activity. Indeed, the nuclear pool of GSK3β appeared more active in OGT null cells and inhibition of GSK3β partially recovered HSP70 protein abundance (87).

The promoter of HSP70 contains binding sites for transcription factors other than HSF1, such as the essential and ubiquitous Sp1 (89). Several studies have demonstrated that Sp1 is modified by O-GlcNAc (90, 91). Treatment of mouse embryonic fibroblasts with glutamine, which enhances UDP-GlcNAc pools and in turn O-GlcNAc (Fig. 2), promotes the nuclear localization of Sp1 and HSP70 synthesis (88). Independent studies demonstrated that O-GlcNAcylation of Sp1 promotes thermal stability, suggesting that during conditions that destabilize Sp1 such as heat shock, O-GlcNAcylation of Sp1 promotes chaperone expression by maintaining the pool of functional Sp1 (92). The impact of O-GlcNAc on the stability of proteins is not unique to Sp1 and will be discussed in greater detail below.

One aspect of the cellular stress response is inhibition of cap-dependent translation and subsequent sequestration of mRNA and ribonucleoproteins in stress granules (93). O-GlcNAc has been implicated in the formation and regulation of stress granules (94, 95), as well as the switch from cap-dependent to cap-independent translation that favors the synthesis of chaperones (96, 97). Eukaryotic mRNAs are modified by a 5′ 7-methylguanosine cap. Recognition of the cap by the eukaryotic initiation factor (eIF) 4F complex, composed of eIFs 4E, 4A, and 4G1, recruits the preinitiation complex. Subsequently, the interaction between the poly(A) binding protein (PABP) and eIF4G1 results in circularization of the mRNA and scanning for start codons (98). Cellular injury inhibits cap-dependent translation using multiple mechanisms including phosphorylation of eIF2α (a component of the pre-initiation complex) and binding of eIF4E by the eIF4E-binding protein 1 (4E-BP1), which blocks its association with eIF4G1. Cap-independent translation requires elements in the 5′UTRs that include internal ribosomal entry sites (98). While the interplay between cap-independent translation and O-GlcNAc on chaperone abundance has not directly been studied in models of injury, recent studies in a model of diabetes support a role for O-GlcNAcylation of 4E-BP1 in the response of cells to hyperglycemia (96, 97). Dennis and co-workers demonstrated that there is a switch to cap-independent translation in conditions in which O-GlcNAcylation of 4E-BP1 is enhanced. Hyperglycemia, glucosamine, or OGA inhibition (thiamet-G; TMG; Fig. 2) promote the association between 4E-BP1 with eIF4E thus inhibiting cap-dependent translation (96). In an extension of these studies, RNA-Seq and ribosomal profiling demonstrated that in mice treated with an OGA inhibitor (TMG; Fig. 2), the mRNAs associated with the ribosome undergo major remodeling, which was prevented by deletion of 4E-BP1 (97).

Glucose starvation is another model in which translation is inhibited. mTORC1 can control translation by phosphorylating 4E-BP1, resulting in the release of eIF4E and an increase in global translation rates. Recently, glycosylation of leucyl-tRNA synthetase 1 (LARS1) was demonstrated to play a role in regulating mTORC1 during glucose starvation. Like other studies (15, 43), Kim and colleagues detect a paradoxical increase in O-GlcNAc globally upon starvation and at Ser1042 of LARS1 (99). O-GlcNAcylation of LARS1 induced a confirmational change, blunting the interaction with the RagD GTPase. Consequently, LARS1 is phosphorylated by Unc-51-like autophagy-activating kinases 1 (ULK1) in its leucine binding site, preventing activation of mTORC1. This signaling axis retunes metabolism during glucose starvation, shifting leucine toward catabolic pathways. Critically, mutation of LARS1 at Ser1042 resulted in constitutive activation of mTORC1 (99).

Independent studies have demonstrated that in response to heat shock, eIF4G1 is O-GlcNAc modified at Ser68 (95). O-GlcNAcylation of the N-terminal domain of eIF4G1 inhibits binding to PABP1, repressing translation (95). Unexpectedly, the authors demonstrated that the mRNA of HSP70, *HSPA1A*, was bound to nonglycosylated eIF4G1. Using a combination of eIF4G1 mutants, the authors provide support for a model in which the association of eIF4G1 and PABP1 regulate stress granule dynamics. During recovery from heat shock, O-GlcNAcylation of eIF4G1 promotes the dissolution of stress granules, which is followed by cap-independent translation of mRNAs such as *HSPA1A,* impacting the abundance of HSP70 and other chaperones. Collectively, these data highlight numerous points at which the cell can regulate transcription and translation to impact chaperone abundance *via* protein O-GlcNAcylation.

##### Chaperone function

O-GlcNAc–targeted glycoproteomic studies demonstrate an enrichment of proteins that play roles in protein quality control, including molecular chaperones (for instance, the chaperonin-containing T-complex) and proteasomal proteins (56, 78, 79, 80). Indeed, independent studies have demonstrated that the small ATP-independent chaperone HSP27 is modified by O-GlcNAc (100, 101, 102). HSP27 impacts cell survival by regulating key players in the apoptotic cascade and by binding partially or fully misfolded proteins, preventing the formation of toxic aggregates (103). Several studies implicate O-GlcNAc in regulating HSP27 abundance and phosphorylation (104, 105); however, the most direct evidence for the impact of O-GlcNAc on HSP27 function is presented in studies using glycoprotein engineering (106, 107, 108). HSP27 contains an N-terminal domain important for oligomerization, a central α-crystallin domain that interacts with client proteins, and a C-terminal domain that contains an IXI/V motif (IPV) (109). The latter domain, which reversibly binds to and inhibits the α-crystallin domain, is O-GlcNAcylated adjacent to the IPV sequence at Thr184 (100, 101, 102). Three studies demonstrate that O-GlcNAcylation of HSP27 impacts chaperone activity and regulation of programmed cell death *in vitro* (106, 107, 108).

Glycoprotein engineering was used to generate constitutively O-GlcNAcylated HSP27 at Thr184 and the related O-GlcNAcylated chaperones (110, 111, 112, 113): α-crystallin at Ser 162 and β-crystallin at Thr162. While unmodified chaperones reduce the aggregation of α-synuclein, glycosylation of all three small chaperones promoted their activity (108). Underpinning enhanced activity of glycosylated HSP27 was a reduction in the association of IPV with the crystallin domain. Furthermore, O-GlcNAcylation enhanced oligomerization of HSP27, potentially improving the efficacy of the chaperones in preventing aggregate formation further (108). As HSP27 binds caspase 3, thus inhibiting activation of caspase 9 and apoptosis, the aforementioned studies were extended to assess the antiapoptotic activity of glycosylated HSP27 (106). Engineered HSP27 O-GlcNAcylated at Thr184 was shown to reduce the rate of cleavage of caspase 3 and inhibit the cleavage of caspase 9 by caspase 3 (106).

Collectively, these data suggest that O-GlcNAcylation of HSP27 may impact diseases of protein misfolding. Confoundingly, the impact of O-GlcNAc on HSP27 function is impacted differentially by mutants that underpin Charcot-Marie-Tooth type 2 disease (107). Notably, O-GlcNAcylation of HSP27 at Thr184 reduces chaperone activity of mutants Thr180Ile, Ser187Leu, and Arg188Trp, whereas glycosylation at Thr184 promotes the chaperone activity of HSP27 carrying either Pro182Leu or Pro182Ser (107). Like the impact of O-GlcNAc on chaperone activity, glycosylation of the mutants differentially impacts oligomerization of HSP27. Notably, larger order aggregates of HSP27 Pro182Leu and Pro182Ser were rescued by O-GlcNAcylation, suggesting a model by which O-GlcNAc promotes the chaperone activity of these proteins by reducing aggregation. In contrast, O-GlcNAcylation of Thr180Ile, Ser187Leu, and Arg188Trp destabilized oligomer reducing their efficacy against unfolded protein (107).

#### The integrated stress response

Studies indicating that XBP1s targets O-GlcNAcylation and that O-GlcNAc regulates cap-dependent translation, implying a role for O-GlcNAc in regulating the cellular response to ER stress and the integrated stress response (ISR) (76, 97, 114). The ISR plays a critical role in maintaining cellular homeostasis by regulating translation, lowering energy consumption, and reprogramming gene expression (115). Activation of four critical kinases by viral infection (protein kinase R; PKR), heme deprivation, and oxidative stress (heme-regulated eukaryotic initiation factor eIF-2-alpha kinase; HRI), ER stress (PRKR-like ER kinase; PERK), and amino acid starvation and UV irradiation (eIF-2-alpha kinase GCN2; GCN2) result in the phosphorylation and inhibition of eIF2α, a subunit of eIF2 (115). Inhibition is reversed by CReP (protein phosphatase 1 regulatory subunit 15B) and GADD34, which combine with protein phosphatase 1, to tune eIF2α phosphorylation. While eIF2α-phosphorylation inhibits global translation, it also elicits preferential translation of mRNAs that potentiate the ISR such as *ATF4*, *CHOP*, *GADD34*, and *4E-BP1* (115). Consistent with ER-stress enhancing GFAT1 abundance, activation of ATF4 has also been demonstrated to target *GFAT1* abundance. In a model of pressure overload induced hypertrophy, hearts overexpressing NADPH oxidase (Nox, Nox-TG) demonstrated increased flux into the HBP and O-GlcNAcylation in both unstressed and stressed conditions. In the Nox-TG hearts, ATF4, OGT, and GFAT1 were elevated. Critically, silencing of ATF4 in cardiomyocytes blunted O-GlcNAc and GFAT1 abundance (116). Nox has also been implicated in a cellular model of hypertrophy, where Nox suppression mitigates the impact of OGT inhibition (OSMI; Fig. 2) on p38 MAP kinase activation and HSP27 phosphorylation (105). While activation of the ISR targets O-GlcNAcylation, there is also evidence that O-GlcNAc regulates key proteins in this pathway. In addition to 4E-BP1, in neuronal cells GFAT1 gain-of-function mutations led to phosphorylation of PRKR-like ER kinase and eIF2α, as well as ATF4 activation (117). Lastly, eIF2α phosphorylation can be protected by the eIF2-associated glycoprotein p67 (118). p67 is glycosylated in conditions such as serum starvation (119, 120).

#### Protein aggregation

Proteomic studies highlight that O-GlcNAc is cycled on and off proteins and sites at different rates (56, 79, 121, 122). One interpretation of these data is that cycling rates imply different functional roles for O-GlcNAc, with low turnover sites playing structural roles and fast turnover sites supporting dynamic regulation. One example of the former may be stabilization of protein structure and in turn half-life. Early studies focused on Sp1 demonstrated that reduced global O-GlcNAcylation was associated with enhanced proteasomal susceptibility (90). Similar observations have been made with nuclear pore protein p62 (Nup62) and 93 (Nup93) (123). Notably, deletion or inhibition of OGT was shown to enhance ubiquitination of Nup62 and degradation *via* the proteosome (123). Mutagenesis of Nup62 at Thr373 and Ser468 confirmed that reducing O-GlcNAc reduced the half-life of Nup62 in a ubiquitin/proteosome-dependent manner (123). Subsequently, Nup62 and Sp1 were shown to be cotranslationally O-GlcNAc-modified and that reducing glycosylation reduced protein abundance. Critically, this observation was not extended to F-box only protein 22 and clusterin, suggesting that this phenotype did not result from inhibition of transcription or translation (124). Nascent Sp1 and Nup62 were found to be more O-GlcNAcylated than their mature counterparts. Zhu and co-workers provide support for a model in which intrinsically disordered regions, prone to cotranslational degradation, are stabilized by O-GlcNAcylation (124). Subsequently, a combination of tools was used to identify an additional 176 proteins cotranslationally O-GlcNAcylated, which included ataxin-1–like protein, nuclear pore protein 152, and host cell factor 1 (125).

Synthesis of O-GlcNAc–modified peptides (126, 127) and proteins (128, 129, 130, 131, 132, 133) highlights the potential of O-GlcNAc to impact protein structure. The role of O-GlcNAc preventing Tau and α-synuclein aggregation, and the subsequent impact on the progression of neurodegenerative disease, is discussed in detail in another review in this series (134). However, thermal shift assays suggest that the impact of O-GlcNAc on protein stability is relevant to proteins outside the central nervous system. The nucleotide-binding oligomerization domain-containing protein 2 (Nod2), whose mutation contributes to Crohn’s disease, is O-GlcNAcylated (135). Like Nup62 and Sp1, elevating O-GlcNAcylation extends the half-life of Nod2. The impact of O-GlcNAc in stabilizing Nod2 can be recapitulated using thermal shift assays, in which the amount of soluble protein is assessed across a temperature gradient (136). A proteome-wide application of this approach was used in tissues treated with a hexosaminidase to reduce O-GlcNAcylation. Consistent with the literature, O-GlcNAc did not stabilize all the detected proteins (137). Interestingly, proteins were found to be both stabilized and destabilized by a decrease in O-GlcNAc. One group of proteins stabilized by O-GlcNAc was those from the proteasome (137).

#### Protein degradation *via* autophagy

Autophagy, or self-eating, is a critical housekeeping process that removes damaged and unwanted organelles and protein aggregates *via* the lysosome (138, 139, 140). Mirroring stress-induced changes in O-GlcNAcylation, autophagy is induced by numerous stimuli that include heat stress, starvation, reactive oxygen species (ROS), hypoxia, ER-stress, lipopolysaccharide, radiation, viral infection, exercise, and IPC. While unregulated autophagy has been associated with maladaptive pathologies such as heart failure, the induction of autophagy is generally considered a prosurvival response of cells (138, 139, 140). Several studies have reported that key proteins involved in autophagy are O-GlcNAcylated and that O-GlcNAcylation appears to both promote and inhibit steps in autophagy (141, 142, 143, 144, 145, 146, 147, 148, 149, 150) (Fig. 3). These counterintuitive observations are best illustrated by studies using *C. elegans* in which deletion of OGT or OGA results in the induction of the autophagy marker LC3-II (PE-LGG-1-GFP) (141). Based on these and other data, we posit that O-GlcNAc cycling regulates different nodes within the autophagic cascade that are further tuned by the stimuli, cell/tissue type, and the amplitude, duration, and method of O-GlcNAc manipulation.

**Figure 3:**
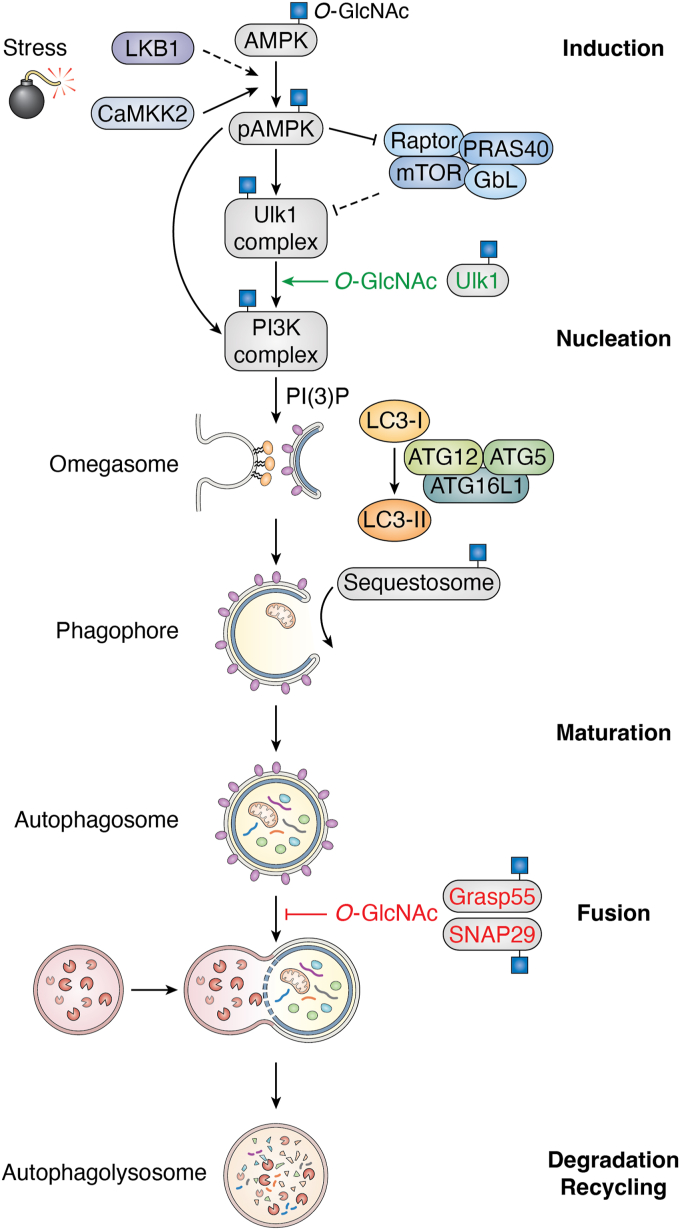
**An overview of the autophagy pathway**. Autophagy can be broken down into five steps: induction, nucleation, maturation, fusion, and degradation. The first committed step of autophagy is activation of Ulk1, which results in the formation of a complex with Atg13, Fip200, and Atg101. In turn, the ULK1 complex can activate the PI3 kinase complex (VPS34, VPS15, Beclin1, ATG14). The VPS34 complex generates phosphatidylinositol (3,4,5)-trisphosphate (PIP_3_) on the cytosolic face of the ER membrane to form the omegasome, which recruits effector proteins WIPI2/DFCP1. In turn, WIPI1/DFCP1 recruits a ubiquitin-like conjugation system, ATG12-AT5-ATG16L1, that cleaves LC3-1 and subsequently conjugates it with phosphatidylethanoloamine to form LC3-II. The latter is critical for the elongation and closure of the phagophore. Several cargo proteins, including p62/sequestosome, recruit cargo to the growing phagophore. Ultimately, the autophagosome fuses with the lysosome, resulting in the degradation of contents. Many proteins within this pathway are O-GlcNAc–modified (156), including central players indicated with a *blue square* (): AMPK (249), ULK1, Beclin1, and p62/sequestosome (56). Steps promoted and inhibited by O-GlcNAcylation are identified. AMPK, 5′-AMP-activated protein kinase; ATG, autophagy; CaMK II, calcium/calmodulin-dependent protein kinase kinase 2; DFCP1, double FYVE-containing protein 1; GRASP55, Golgi reassembly-stacking protein of 55 kDa; LC3, microtubule-associated proteins 1A/1B light chain 3B; LKB1, liver kinase B1; mTOR, mammalian target of rapamycin; PIP_3_, phosphatidylinositol-3,4,5-triphosphate; SNAP29, synaptosomal-associated protein 29; ULK1, Unc-51–like kinase 1; WIPI1, WD repeat domain phosphoinositide-interacting protein 1.

The first committed step of autophagy is activation of ULK1 (Atg1) (Fig. 3). Activation of Ulk1 results in the formation of the mATG13–Fip200–Atg101 complex and in turn the PI3K complex (VPS34, VPS15, Beclin1, ATG14). Ulk1 is modified by numerous PTMs including acetylation, phosphorylation, methylation, ubiquitination, and O-GlcNAcylation at Thr754 (139). In a liver model of starvation, Ruan and co-workers demonstrated that withdrawing nutrients led to an elevation of O-GlcNAc in cell culture and animal models (145). In parallel, autophagy was activated as evidenced by turnover of p62/sequestosome and the accumulation of LC3-II. Treatment of cells with an OGA inhibitor (TMG, Fig. 2), or overexpression of OGT, recapitulated these data, suggesting that cellular O-GlcNAcylation promoted autophagy (145). Consistent with these observations, an inducible OGT knockdown in the liver blunted the induction of autophagy in response to fasting. Critically, starvation induced the O-GlcNAcylation of ULK1. Mutation of known O-GlcNAcylation sites at Thr635 and Thr754 (151, 152) reduced O-GlcNAcylation and association with AMPK, while modestly reducing Ulk1 activity toward Beclin-1 (145). Subsequently, an additional O-GlcNAcylation site on Ulk1 was identified at Ser409. Mutation of this site to alanine renders ULK1 less stable, which may result from enhanced destabilizing phosphorylation at Ser423 (153). Critically, mutation of Ser409 reduced Ulk1 association with the lysosome and association with syntaxin-17 (STX17). The latter plays a critical role in promoting autophagosome/lysosome fusion, suggesting that O-GlcNAc potentiates autophagy in a model of human papillomavirus infection by stabilization of ULK1 and subsequent turnover of autophagosomes (153). O-GlcNAcylation of ULK1 has also been reported to impact hepatitis B virus (HBV) replication (154). O-GlcNAcylation and OGA abundance are reported to decrease upon HBV infection. In contrast to the two prior studies, lowering O-GlcNAcylation *via* OGT inhibition (OSMI-1, Fig. 2) or short-interfering OGT RNAs induced autophagy in a model of HBV infection. Induction of autophagy was associated with increased Ulk1 protein abundance and inhibition of mammalian target of rapamycin (mTOR) (154).

One mechanism by which cells suppress ER stress is by upregulating ER-phagy, the degradation of ER-derived vesicles *via* autophagy (155). Recent studies in a model of intervertebral disc degeneration (IDD), demonstrate that family with sequence similarity 134 member B (FAM134B; RETREG1) protein abundance, and in turn ER-phagy, is impacted by O-GlcNAcylation. In the nucleus pulposus of patients with severe IDD, O-GlcNAcylation and OGT abundance are enhanced on cellular proteins (147). Like models of acute stress, OGT overexpression depressed the appearance of apoptotic markers in IDD (for instance, cleaved caspase 3) (147). One critical step in ER-phagy is ER fragmentation at autophagosome biosynthesis sites, which requires the association of atlastin 2 with FAM134B (155). Overexpression of OGT increased the abundance and colocalization of FAM134B and LC3-II, suggesting that ER-phagy was enhanced in response to nutrient deprivation. Critically, the impact of OGT overexpression on markers of ER-phagy was reduced in the absence of FAM134B (147). FAM134B half-life was extended in cells treated with inhibitors of OGA (TMG, Fig. 2), whereas inhibition of OGT (OSMI-1; Fig. 2) reduced FAM134B half-life. Suggesting a mechanism for these data, FAM134B was shown to be O-GlcNAcylated and to associate with OGT (147, 156).

Recent studies suggest that O-GlcNAc may also play a role in mitophagy or the removal of aged or damaged mitochondria by autophagy (157). Ubiquitin-dependent mitophagy is driven by Pink1, which is imported into the mitochondria and degraded under normal conditions. Mitochondrial damage leads to the accumulation and dimerization of Pink1 (157). Subsequently, activation of Pink1 leads to phosphorylation of ubiquitin and the ubiquitin-like domain of PARKIN, which in turn recruit adapter proteins such as p62/sequestosome, optineurin, and calcium-binding and coiled-coil domain-containing protein 2 (NDP52) (157). Disruption of OGT in hematopoietic stem cells results in the accumulation of ROS, a result of mitochondrial dysfunction (158). RNA-seq analysis revealed reduced expression of *PINK1*. Suggesting a defect in mitophagy, colocalization of PARKIN with mitochondria was decreased in OGT-deficient hematopoietic stem cells. Ultimately, OGT was demonstrated to regulate transcriptional activation of *PINK1* by regulating histone methylation (144, 158).

In contrast to the data discussed above, O-GlcNAc has also been demonstrated to blunt autophagy (Fig. 3). Fusion of autophagosomes with lysosomes requires the concerted action of RAB small GTPases, tethering factors, and synaptosomal-associated protein (SNAP) receptor (SNARE) complexes (159). Critical to this discussion is the SNARE complex composed of STX17, SNAP29, and vesicle-associated membrane protein 8 (VAMP8). Interaction of the RAB7 effector ectopic P-granules protein 5 homolog with LC3 facilitates the formation of the STX17–SNAP29–VAMP8 SNARE complex, thus promoting autophagosome maturation. Guo and co-workers used a suppressor screen to identify genes in *C. elegans* that blunted the accumulation of p62/sequestosome in an ectopic P-granules protein 5 mutant background (160). Using this screen, OGT was identified as a suppressor of p62/sequestosome accumulation. As deletion of *OGT* in *C. elegans* and mammalian cells led to autophagic flux, these data suggested that OGT is a suppressor of autophagy (160). Notably, the association between SNAP29 and VAMP8 was elevated in *OGT* null cells, suggesting that reducing O-GlcNAcylation promoted formation of the SNARE complex and subsequent autophagosome maturation. Indeed, SNAP29 was demonstrated to be multiply O-GlcNAcylated and that mutation of these sites promoted autophagosome-lysosome fusion (160). Subsequently SNAP29 was demonstrated to be O-GlcNAcylated in cells treated with arsenite, a treatment that also inhibited autophagy. Consistent with the work of Guo and co-workers (160), an O-GlcNAcylation defective SNAP29 abolished arsenite-mediated autophagy inhibition (144).

The Golgi apparatus is a polarized stack of closely opposed and flattened cisternae that play critical roles in protein glycosylation and trafficking. Adjacent cisternae are stabilized by proteins, including Golgi reassembly stacking protein (GRASP) 65 kDa (GRASP65) and 55 kDa (GRASP55) (161). Recently, GRASP55 was demonstrated to be O-GlcNAcylated (162). Glucose starvation induces the relocalization of GRASP55 from the Golgi to puncta, which colocalize with LC3. GRAP55 is basally O-GlcNAcylated, and in response to glucose starvation, O-GlcNAcylation is reduced (162). Mutational analysis demonstrated that substitution of five serine and threonine residues with alanine (5A mutant: Ser389Ala, Ser390Ala, Thr403Ala, Thr404Ala, and Thr413Ala) significantly reduced glycosylation of GRASP55. Deletion of GRASP55 resulted in autophagosome accumulation, suggesting that GRASP55 plays a role in autophagosome/lysosome fusion (162). As the 5A mutant rescued autophagy to a larger extent than WT GRASP55, the authors conclude that O-GlcNAcylation of GRASP55 inhibits lysosome/autophagosome fusion. Underpinning these observations, the 5A mutant of GRAP55 has enhanced interactions with LC3 and the lysosome-associated membrane glycoprotein 2 (162). As amino acid withdrawal and mTOR inhibition also led to GRASP55 deglycosylation, these data suggest that loss of O-GlcNAc on GRASP55 in response to nutrient deprivation upregulates autophagy (162).

### O-GlcNAc and the cardiovascular system

The cardiovascular system is complex and dynamic in its response to physiologic and pathologic stimuli. Myriad inputs are translated to molecular signals for adaptation at the cellular and cardiac tissue level. O-GlcNAcylation has emerged as a powerful and potent transducer of biological inputs to the heart. OGT, OGA, and the HBP play important roles that govern O-GlcNAcylation and thereby regulate key cardiac processes including transcription, translation, cellular energetics, excitation-contraction coupling, and cell death (13, 163, 164). Decades of research exploring the role of O-GlcNAc biology in the cardiovascular system support the idea that O-GlcNAcylation has an essential role in cardiovascular physiology and that O-GlcNAc biology has an equally important, and complex, role in cardiovascular disease (13, 163, 164). Here, we endeavor to highlight seminal studies that have advanced our understanding of O-GlcNAc and the cardiovascular system (Fig. 4).

**Figure 4:**
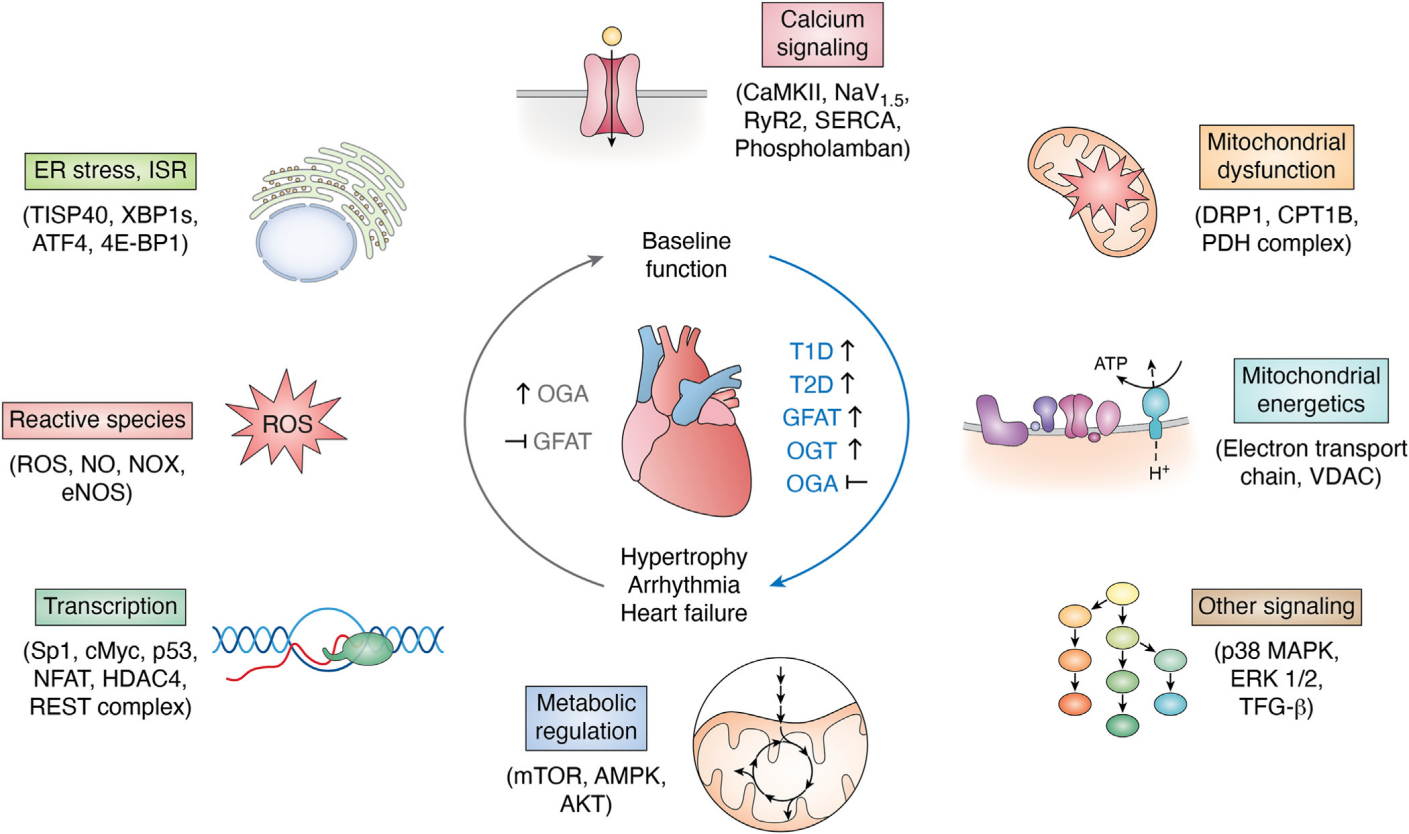
**The impact of sustained changes in cardiac O-GlcNAcylation**. Pressure overload, diabetes, and other heart derangements lead to elevated O-GlcNAcylation that is associated with maladaptation. Pathologic phenotypes can be recapitulated by genetic and pharmacological upregulation of O-GlcNAc (indicated in *blue*) and counteracted by blocking the HBP or reducing O-GlcNAcylation (indicated in *gray*). Changes in O-GlcNAc are associated with pathology *via* changes in transcription, reactive oxygen and nitrogen species, calcium handling, ER stress, signal transduction, and mitochondrial energetics and function. ER, endoplasmic reticulum; HBP, hexosamine biosynthetic pathway.

#### Cardiac cell biology

Salient cell types in the heart include cardiomyocytes, endothelial cells, and fibroblasts. The interactions between these cells determine the vital functions of the heart. O-GlcNAc biology has been studied in all these cell types, where it plays important roles.

##### Acute changes in O-GlcNAcylation

Much of the early work to understand O-GlcNAc biology was conducted in neonatal cardiac myocytes subjected to short exposures to stress (*i.e.,* hypoxia and simulated ischemia) or elevation in O-GlcNAcylation *via* modulation of glucose, the HBP, OGT, or OGA (Fig. 2). As described above, acute elevation of O-GlcNAcylation was correlated with the induction of cardiac cytoprotective pathways. For instance, increased O-GlcNAcylation achieved by treating cells with glucosamine and/or an OGA inhibitor (PUGNAc, Fig. 2) before inducing injury improved cellular viability (83, 84, 165). Cytoprotection induced by O-GlcNAcylation was associated with preserved mitochondrial membrane potential and reduced ROS levels, calcineurin activation, and reduced ER stress (83, 84, 166). Similarly, adenoviral OGT overexpression increased cardiomyocyte survival after hypoxia (167). Interestingly, decreased OGT expression results in lower basal O-GlcNAc levels and was noted to confer increased cellular injury in cardiomyocytes exposed to I/R injury. While lowering O-GlcNAc levels did not impact Bcl-2 abundance, translocation of Bcl-2 to the mitochondria (with presumed antiapoptotic effects) in response to I/R injury was blunted (165).

##### Chronic changes in O-GlcNAcylation

Observations in cultured cells and animals exposed to prolonged stress (type 1 or 2 diabetes (T1D or T2D), hypertension) have also been used to understand the role of O-GlcNAcylation and molecular changes in the cardiomyocyte. Cardiac myocytes exposed to a chronic high glucose treatment have increased mitochondrial O-GlcNAcylation and impaired activity of complex I, III, and IV (168, 169). Lower mitochondrial calcium and cellular ATP content in this model was ameliorated by OGA overexpression, which was accompanied by improved mitochondrial function (168, 169, 170). Altered O-GlcNAcylation impacts cardiomyocyte hypertrophy and signaling through salient hypertrophic pathways such as AKT and extracellular signal-regulated kinase 1/2 (ERK 1/2) (65, 105). GFAT2 abundance is increased in response to several hypertrophic stimuli, including isoproterenol (Fig. 2, above) (65). Knockdown or inhibition (diazo-5-oxo-L-norleucine [DON], Fig. 2) of GFAT2 suppresses isoproterenol-induced cardiomyocyte hypertrophy and is accompanied by suppression of Akt O-GlcNAcylation and signaling (65). Conversely, increasing protein O-GlcNAcylation was accompanied by Akt activation and cardiomyocyte hypertrophy. Other studies posit an important role of O-GlcNAcylation for maintaining balanced activity of Erk1/2 and p38 MAP Kinases during hypertrophic growth responses in cardiomyocytes (105).

##### Fibroblasts

An important cell type in the heart is the cardiac fibroblast. These cells produce and secrete growth factors, cytokines, and other signaling molecules that serve an important role in paracrine signaling. Cardiac fibroblasts overexpressing OGA show decreased total protein O-GlcNAcylation with a concomitant reduction in transforming growth factor-β1 and SMAD 2/3 protein abundance (171). Interestingly, these changes correlated with a decrease in the O-GlcNAcylation of the transcription factor Sp1 (90) and inhibitory effects on profibrotic signaling *via* collagen I and III protein levels (171).

#### Cardiac physiology

##### Vascular biology

Sustained alteration of cardiovascular reactivity can result in hypertension, a primary risk factor for cardiovascular disease (172). There are notable effects on the vascular endothelium after both exposure to hyperglycemia and increased shear stress (173, 174, 175, 176). Rat aortic smooth muscle cells exposed to hyperglycemia display increased OGT abundance and O-GlcNAc levels (173). In vascular smooth muscle cells, excess HBP flux from either hyperglycemia or glucosamine treatment results in increased O-GlcNAcylation of Sp1, thereby attenuating its degradation and influencing the regulation of multiple genes, including, profibrotic gene programs (90). Hyperglycemia-mediated increases in OGT and O-GlcNAc are associated with downregulation of miR-200a/200b (176), a class of microRNA with links to aging, diabetes mellitus, and arrhythmia (177). Interestingly, attenuation of O-GlcNAcylation has been reported to be beneficial in certain hyperglycemic conditions. In a T2D mouse model, endothelial cell-specific overexpression of OGA improved coronary microvascular function by reducing an increase in p53 abundance (178). In a T1D mouse model, endothelium-dependent induced overexpression of OGA reduced endothelial O-GlcNAc levels and restored endothelial-dependent vasodilation. This may, in part, be due to O-GlcNAcylation being strongly associated with the regulation of the potent vasodilator, endothelial nitric oxide synthase (eNOS). Increased expression of endothelial OGA in the diabetic group did not significantly decrease eNOS O-GlcNAcylation; however, there was significant reduction in connexin-43 O-GlcNAcylation, suggesting that this might contribute to the improved vascular function (178).

Hyper-O-GlcNAcylation is linked to vascular dysfunction in models other than diabetes. In the deoxycorticosterone acetate and salt hypertensive rat model, O-GlcNAc levels are elevated in arteries after 5 weeks with a concomitant increase in blood pressure and enhanced vasoconstriction in response to phenylephrine (179, 180). Inhibiting OGA (PUGNAc, Fig. 2) in normotensive animals also enhanced vasoreactivity, suggesting that O-GlcNAc plays a pathologic role. This may, in part, be due to O-GlcNAcylation being strongly associated with the regulation of the potent vasodilator, eNOS. Phosphorylation of eNOS, Akt, and PI3K were all decreased in hypertensive animals, whereas eNOS O-GlcNAcylation was increased (179, 180). Vascular function studies demonstrated impaired endothelin-1–dependent relaxation and enhanced sensitivity to vasoconstrictors. Augmenting O-GlcNAcylation (PUGNAc; Fig. 2) was sufficient to recapitulate these vascular effects in normoglycemic conditions, indicating a role for O-GlcNAc in the development of hypertension that was independent of circulating glucose tension (181). These and other studies, led Lima and co-workers to suggest a model in which endothelin-1 stimulates O-GlcNAcylation, leading to increased vascular contractile responses *via* activation of the RhoA/Rho-kinase pathway (182).

As delineated above, O-GlcNAcylation appears to have a biphasic role transducing hyperglycemia, shear stress, and inflammation to the vasculature impacting reactivity and behavior. This may, in part, be due to the interplay between direct regulation of master signaling regulators by O-GlcNAc and regulation of upstream and downstream pathways. For instance, O-GlcNAcylation of eNOS at Ser1177 blocks its activation by AKT in a model of hyperglycemia (174). eNOS activity also appears to be reduced by direct O-GlcNAcylation at Ser615 (183). Vascular dysfunction has also been associated with direct O-GlcNAcylation of AKT. Heath and co-workers proposed that chronic inhibition of OGA (TMG; Fig. 2) accelerated the development of vascular calcification *via* increased O-GlcNAcylation of AKT (184). In contrast to these data, OGA inhibition (TMG, Fig. 2) or glucosamine treatment of aortic rings protects from endothelial dysfunction and diminished contractility induced by tumor necrosis factor α (185). Additional studies in this model led the authors to conclude that O-GlcNAcylation of nuclear factor kappa-B p65 inhibits acute inflammatory response that aggravate endoluminal injury (186, 187).

#### Physiologic hypertrophy

Physiological hypertrophy is considered an adaptive, compensatory response that commonly occurs following chronic exercise training. In contrast to pathological hypertrophy, physiological hypertrophy is an adaptive response that has not traditionally been thought to progress to heart failure (188). Swim-trained mice show a global decrease in cardiac O-GlcNAcylation when compared to sedentary animals. This observation correlates with a reduction in *GFPT1/2* mRNA, as well as a significant decrease in *OGT* mRNA and OGT protein abundance (189). Similarly, a single and short period of treadmill exercise results in a reduction of cardiac cytosolic O-GlcNAcylation when compared to untrained mice. In this model, OGT is proposed to interact with the repressor element 1–silencing transcription factor (REST) (190). REST acts as a chromatin repressor by interacting with corepressors and class II histone deacetylases (HDACs). Acute exercise resulted in dissociation of OGT from the REST complex and mSin3a, triggering physiological hypertrophy signaling (190). These studies also suggest that in the mouse heart HDACs 1, 2, 4, and 5 are targets of O-GlcNAcylation, which might reinforce changes in hypertrophic signaling (190). More recent work highlights another important protein in hypertrophic transcriptional machinery, stromal interaction molecule-1. Stromal interaction molecule-1 is an O-GlcNAc target and O-GlcNAcylation is proposed to attenuate store operated calcium entry in cardiomyocyte (191).

O-GlcNAcylation is an important transducer of energetic signatures in the heart and elevated O-GlcNAcylation is linked to AMPK and eNOS activity (192). Gélinas and co-workers proposed a model in which AMPK inhibits O-GlcNAcylation by inhibiting GFAT *via* phosphorylation, thereby reducing O-GlcNAcylation of proteins including troponin T, a component of the contractile complex in the heart. As prohypertrophic stress (angiotensin II) increases AMPK and O-GlcNAcylation, this bidirectional relationship improves heart function and limits cardiac hypertrophy by attenuating HBP flux (193). Understanding that cardiac energetics, metabolism, and hypertrophy are closely coupled; it is unsurprising that significant differences are observed in the cardiac O-GlcNAcylation level of several mitochondrial proteins (167, 194). O-GlcNAcylation levels of complex I, complex IV proteins, the voltage-dependent anion channel, and SERCA are higher in a low running capacity group of rats when compared to high running capacity group rats (195). Additionally, several prohypertrophic transcription factors are regulated by O-GlcNAcylation. The transcription factors, Sp1 and c-Myc (90, 171, 196), have been shown to have multiple O-GlcNAcylation sites and are involved in regulation of many cardiac genes, including those involved in cardiomyocyte hypertrophy (171, 195).

#### Cardiac pathology

##### Cardiac hypertrophy

Pressure overload-induced cardiac hypertrophy is encountered clinically in the form of increased afterload, commonly hypertension, and valvular disease and is defined by a change in metabolic substrate preference that is concomitant to the structural remodeling of the heart (197). The normal heart preferentially uses fatty acids to produce the ATP required for its function, whereas hypertrophied heart utilizes mainly glucose (197). Cardiac remodeling during hypertrophy is characterized by the altered expression of many proteins and reversion to the fetal gene program (switch from alpha-myosin heavy chain to the beta-myosin heavy chain) and can be influenced by metabolic switch, favoring glucose uptake instead of fatty acids (198). Isoform switches occur in nonhypertrophic hearts forced to consume glucose (198). The observed switch has been linked to *GFPT2* mRNA levels, suggesting that O-GlcNAcylation of proteins could be part of this mechanism (65).

Several studies have demonstrated changes in O-GlcNAc, OGT, and OGA abundance with respect to the development of pathologic hypertrophy in the setting of cardiovascular stress (46, 189, 199, 200). A cardiomyocyte specific inducible OGT null mouse demonstrates an increase in cardiomyocyte hypertrophy in a model of trans aortic construction (TAC) (199). Consistent with this observation, constitutive ablation of cardiomyocyte OGT resulted in marked increase in heart weight and increased cardiomyocyte size as well as increased embryonic and perinatal death (201). In contrast, global O-GlcNAcylation was increased in humans, hypertensive rats, and rats after aortic banding (46). The study reported O-GlcNAcylation was increased in myocardium obtained from patients with aortic stenosis, in hypertensive rats, postaortic banding, and post myocardial infarction (MI) in hypertrophic and failing hearts (46). OGT, OGA, and GFAT2 protein and/or mRNA levels were increased by pressure overload, while neither was regulated by MI in this study (46). Our group recently demonstrated increased cardiomyocyte size and pathological cardiac hypertrophy in a cardiomyocyte OGT overexpression model (OGT TG) (200). Critically, we showed that overexpression of OGA in murine myocardium (OGA TG) was not associated with pathologic remodeling and was protective against pathological hypertrophy after TAC (200). Using a more extended timeline, Zhu and co-workers demonstrated that while O-GlcNAc levels are elevated one-week post-TAC, O-GlcNAc levels decline after 6 weeks. Elevation of O-GlcNAc (TMG, Fig. 2), starting 4 weeks after TAC, did not affect cardiac mass further (202).

Several mechanisms are thought to underpin the role of O-GlcNAc in hypertrophy. A proteomics study designed to identify proteins differentially O-GlcNAcylated in pressure overload identified 700 putative O-GlcNAcylated proteins (203). Two hundred thirty-three of these proteins had significantly increased enrichment in pressure overload over sham, suggesting higher O-GlcNAc levels. In contrast, no proteins were significantly decreased by pressure overload. Carnitine palmitoyl transferase B and the pyruvate dehydrogenase complex demonstrated enhanced O-GlcNAcylation in response to pressure overload. Enzyme activity assays suggested higher O-GlcNAcylation increases carnitine palmitoyl transferase activity and decreases pyruvate dehydrogenase complex activity, further underscoring the metabolic link between cardiac energetics and cardiac hypertrophy. Several other proteins have been implicated in potentiating hypertrophy in an O-GlcNAc–dependent manner. c-Myc overexpression induced cardiac hypertrophy and increased O-GlcNAc levels (204); whereas a *MYC* knockout attenuated pressure overload-induced hypertrophy and decreased O-GlcNAc levels (205). Similarly, neonatal cardiomyocytes treated with either high glucose or OGA inhibition showed an increase in O-GlcNAc levels, as well as basal hypertrophy. This hypertrophic response was prevented by OGT silencing (206). Additionally, the study proposes a possible link between O-GlcNAc and cardiac hypertrophy *via* the activation of the prohypertrophic kinase ERK1/2, leading to cyclin D2 expression (206). The transcription factor nuclear factor of activated T cells was inhibited by reducing O-GlcNAcylation (DON, Fig. 2) and prevented phenylephrine-induced hypertrophic responses in cultured cardiomyocytes. These data show that inhibiting the O-GlcNAcylation process is sufficient to block hypertrophy progression (207). Another molecule implied in potentiating hypertrophy is AMPK (192). In WT mice, OGA inhibition (PUGNAc; Fig. 2) and glucosamine treatment reverses AMPK action. As discussed above, the authors propose that AMPK inhibits O-GlcNAcylation by controlling GFAT phosphorylation and in turn O-GlcNAcylation (192).

#### Ischemia and I/R injury

In response to ischemia, O-GlcNAc levels increase briefly before declining (165). In contrast, IPC leads to elevated levels of O-GlcNAc (47, 83). As elevating O-GlcNAcylation before or after I/R injury provides significant cardioprotection in both *ex vivo* and *in vivo* model systems, these data suggest that O-GlcNAc controls cell fate decisions in the myocardium (82, 83, 84, 85, 86, 165, 166, 167, 208, 209). rIPC has been shown to increase myocardial O-GlcNAc levels in human atrial trabeculae and blocking O-GlcNAcylation abrogated the cardioprotective effect of rIPC (48). Critical examples in cell-based models of I/R injury demonstrate that glucosamine and glutamine protect neonatal rat ventricular myocytes, likely *via* O-GlcNAcylation (165). The strongest evidence supporting cardioprotection afforded by increasing O-GlcNAc levels arise from studies that have either directly stimulated or inhibited O-GlcNAc synthesis in cardiomyocytes. An increase of protein O-GlcNAcylation by inhibition of O-GlcNAcase pharmacologically (PUGNAc, Fig. 2) (84, 210) or genetically by RNA interference of OGA (167, 208) was shown to be protective against early, ischemic injury in neonatal rat ventricular myocytes.

Pharmacologic and genetic augmentations of O-GlcNAc levels *in vivo* or in isolated perfused hearts reduced infarct size when subjected to I/R injury (82, 83, 85, 211, 212). The mechanisms for damage in this context are many but feature rapid shifts in calcium flux and oxidative stress. Studies have indicated that increased O-GlcNAcylation may attenuate the initial calcium influx that occurs in reperfusion, thereby reducing the consequences of Ca^2+^ overload (83, 85, 208). In cases of increased O-GlcNAcylation, it has been proposed that there may be an increase in the tolerance of mitochondria to oxidative stress, possibly by direct modification of mitochondrial proteins such as voltage-dependent anion channel (83). O-GlcNAc–mediated protection of mouse embryonic stem cells against the hypoxia-induced apoptosis may occur due to increased expression of glycerol-3-phosphate acyltransferase-1 and subsequent mTOR activation (213). Critically, loss of OGT exacerbates early infarct-induced heart failure (209).

#### Arrhythmia

It has become clear clinically that arrhythmic risk increases in settings of chronic hyperglycemia, systemic inflammation, and oxidative stress. These mechanisms, individually, and additively, contribute to myocardial fibrosis, including in the atria, ventricles, and conduction systems. The structural alterations of the myocardium predispose patients to both atrial and ventricular arrhythmias. Additionally, clinical entities such as myocardial ischemia, MI, and ischemic cardiomyopathy contribute to the increased risk for ventricular arrhythmias (214). Changes in O-GlcNAcylation accompany many of the aforementioned stressors and couple closely with the development of cardiac arrhythmias (Fig. 4).

A mechanism in cardiac arrhythmia development is the dysregulation of the key calcium homeostatic enzyme in the heart: calcium and calmodulin–dependent protein kinase II (CaMKII) (215). In a diabetic model, hyperglycemia is associated with increased cytosolic ROS, which results in CaMKII activation (216). CaMKII has been shown to be O-GlcNAcylated at S280 (S279 in CaMKIIα). Hyperglycemia is reported to increase CaMKII activity *via* increased O-GlcNAcylation of S280 (216). The hyperglycemia-mediated increase in O-GlcNAc levels was linked to an increased susceptibility to arrhythmias in diabetes (216). However, additional data suggests a more complex molecular phenotype. Under high glucose conditions, O-GlcNAcylation was associated with an increase in Ca^2+^ leak through the ryanodine receptor (RyR)2 that persisted after CaMKII inhibition. While CamKII can phosphorylate and activate RyR2 directly, in this model high glucose/O-GlcNAc appeared to increase ROS leading to calcium leaks indirectly. Mutation of Ser280 of CaMKII prevented an O-GlcNAc–induced increase in Ca^2+^ leak (217). In contrast to these data, Mesubi and colleagues report T1D and T2D significantly increased atrial fibrillation (AF) and this increase required CaMKII and O-GlcNAcylation (218). T1D and T2D both required oxidized-CaMKII to increase AF; however, Mesubi and co-workers did not detect O-GlcNAc–modified CaMKII or a role for O-GlcNAc–modified CaMKII in diabetic AF. Their data suggests that CaMKII is a critical proarrhythmic signal in diabetic AF but that O-GlcNAcylation promotes AF by CaMKII-independent mechanism(s) (218). In support of these data, Umapathi *et al*., reported *de novo* ventricular arrhythmias and sudden death in mice overexpressing myocardial OGT. In this model, excess O-GlcNAcylation was correlated with a high frequency of calcium sparks. Notably neither the level of O-GlcNAc nor the frequency of calcium sparks was rescued by inhibition of CaMKII, suggesting that O-GlcNAc impacts arrhythmias *via* multiple pathways (200). Other salient calcium responsive elements involved in arrhythmia modified by O-GlcNAcylation include SERCA (169), phospholamban (219), and RyR 1 and 2 (100, 220, 221).

Apart from the previously mentioned effects on calcium signaling in the heart, O-GlcNAcylation is emerging as a regulator of other voltage gated channels (222). The activation of voltage-gated sodium channels (Na_v_1.5 or Na_v_1.8) facilitate the late sodium current, lead to calcium “sparks” reflecting the opening of the type 2 RyR2, and are a hallmark of atrial and ventricular arrhythmias. These abnormalities have been attributed to O-GlcNAcylation of Na_v_1.5, as they are abolished following inhibition of UDP-GlcNAc synthesis (222). Hyperglycemia increased the O-GlcNAcylation and expression of Na_v_1.5, as well as blunting the interaction between Na_v_1.5 and Nedd4–2/SAP-97. Critically, hyperglycemia resulted in cytoplasmic aggregation of Na_v_1.5, which could be reversed by GFAT inhibition (DON, Fig. 2). In this model, dysregulation of Na_v_1.5 by O-GlcNAc was associated with loss of function of the sodium channel and prolongation of the PR/QT interval. The latter is clinically relevant to the generation of cardiac arrhythmias (222).

#### Heart failure

Over the course of the last 3 decades the role of O-GlcNAcylation appears increasingly to be one of hormesis in the cardiovascular system. Transient bursts of O-GlcNAcylation during short-term stresses produce adaptive responses that limit myocardial injury, whereas sustained “gain” of O-GlcNAcylation during long-term stress influences the cardiac milieu toward cardiac dysfunction and eventual failure (Fig. 4). Heart failure has been associated with increased O-GlcNAcylation and compromised cardiac contractility. This is exemplified by studies demonstrating that driving OGT expression in the myocardium promotes cardiac dysfunction (200), as does GFAT overexpression (223) and deletion of OGA (224).

Recent data suggests a role for O-GlcNAcylation in heart failure with preserved ejection fraction (HFpEF). O-GlcNAcylation impairs NOS (225) and promotes microvascular dysfunction, two salient features of HFpEF that can be ameliorated by overexpression of OGA (226). Derangements in metabolic pathways and cardiac energetics as etiologies of HFpEF continue to emerge (227). Recently, a mouse model expressing a dominant negative OGA was used to increase cardiomyocyte O-GlcNAcylation. An initial reduction of metabolic pathways (2 weeks) was followed by cardiac remodeling (24 weeks), with some features of diastolic dysfunction and increased fibrosis. Consistent with these observations, increased O-GlcNAcylation is associated with increased fibrosis and adaptive remodeling in diabetes (50), with activation of fibroblasts and collagen synthesis associated with both diastolic and systolic dysfunction (171). Other studies have reported phenotypic reversal of fibrosis and dilated cardiomyopathy by decreasing O-GlcNAcylation (228).

The role of O-GlcNAcylation in conjunction with diabetic cardiomyopathy has been studied with a focus on diastolic dysfunction and cardiac fibrosis. Prakoso and co-workers reported elevated O-GlcNAcylation in the myocardium of diabetic patients (229). Using recombinant AAV (rAAV) encoding OGT and OGA in nondiabetic and diabetic mice, they showed rAAV6-OGT was sufficient to impair left ventricular (LV) diastolic function and induce maladaptive cardiac remodeling and cardiac fibrosis. In contrast, rAAV-OGA rescued LV diastolic function and cardiac remodeling in diabetic mice by preserving PI3K-Akt signaling (229). Kronlage and colleagues suggest that O-GlcNAcylation of HDAC4 at Ser642 is cardioprotective in diabetes mellitus and counteracts pathological CaMK II signaling (230). Other studies report that enhanced O-GlcNAcylation inhibits myocyte contractility (231) and phosphorylation of troponin T (232).

Cardiac energetics play a significant role in the development of both heart failure with reduced ejection fraction and HFpEF (233). Several studies have linked increased myocardial glucose utilization to mitochondrial dysfunction *via* enhanced O-GlcNAcylation (170, 234, 235, 236, 237). O-GlcNAcylation can interfere with oxygen consumption and ATP production in cardiomyocytes through several mechanisms, including direct interference with the electron transport chain, enzymes involved in aerobic respiration (50, 237), and with mitochondrial DNA repair (238). Our group showed myocardium specific overexpression of OGT with elevated cardiomyocyte O-GlcNAc levels in mice was associated with impairment in mitochondrial energetics and led to severe heart failure with reduced ejection fraction. In contrast, the myocardial OGA TG lowered O-GlcNAcylation and mitigated pathological hypertrophic stress (200). Enhanced O-GlcNAcylation has been associated with maladaptive mTOR signaling, enhanced AMPK signaling and nuclear factor of activated T cells activation (200, 223, 239).

O-GlcNAcylation has an important role as a common, terminal pathway for many clinical cardiovascular diseases, including pulmonary hypertension and right ventricular failure. In pulmonary hypertension, reducing O-GlcNAcylation partially improves right ventricular function and metabolic signatures (239). Furthermore, recent studies suggest a role for O-GlcNAcylation in pathologies such as Takotsubo cardiomyopathy. This latter model is a stress-induced cardiovascular disease with symptoms comparable to those of an acute coronary syndrome but without coronary obstruction. In an experimental rodent model of stress cardiomyopathy, dysregulation of glucose metabolism and increased O-GlcNAcylation were noted 7 days poststress in the (LV apex (240).

## Conclusions and future directions

From a translational perspective, it is important to recognize that observations made in cardiac myocytes and small animal models have been observed in human myocardium. O-GlcNAcylation is enhanced in the hearts of patients with T2D or aortic stenosis, as compared with healthy controls (46, 229). It appears as heart failure ensues and advances the level of O-GlcNAcylation increases. Remarkably, levels of O-GlcNAcylation revert toward normal even in patients with advanced heart failure, who have been treated with mechanical unloading using a LV assist device (241). In contrast to heart failure, augmenting O-GlcNAc during reperfusion enhances heart function and survival in models of cardiac MI and trauma hemorrhage, respectively (86, 242, 243, 244). Collectively, these findings highlight the essential role of O-GlcNAcylation in the progression of cardiomyopathy and the treatment of MI, underscoring the potential of using this molecular pathway as a therapeutic target.

To realize the potential of O-GlcNAc in clinical models of cardiovascular disease, several advances are required. Firstly, high-quality, validated, O-GlcNAc site-mapping of myocardial proteins. We anticipate that this approach used in a protein-specific or global manner throughout disease progression will identify key proteins and pathways that underpin the switch between protective and maladaptive cardiac phenotypes. Such studies have the additional benefit of identifying key biomarkers that can be used clinically, underpinning the development of tools such as site-specific antibodies, and aiding studies focused on understanding the regulation of OGT and OGA in the heart. Secondly, a greater focus on studying the role of O-GlcNAc on specific proteins *in vitro* and *in vivo* using site-specific mutations and emerging approaches that enable the integration of O-GlcNAc at specific amino acids (128, 245, 246, 247). We anticipate that these approaches will address some of the limitations of manipulating O-GlcNAcylation globally, providing more direct insight into the impact of O-GlcNAc on protein function. Lastly, a better understanding of the mechanisms that regulate O-GlcNAc-cycling in the heart. This insight is critical, as the abundance of OGT and OGA are not always tied to O-GlcNAc levels and recent studies suggest that pools of UDP-GlcNAc are sufficient to support significant changes in O-GlcNAc without enhanced flux of metabolites into the HBP (248). Such studies would include assessments of HBP flux and enzymatic activities, as well as PTMs and protein interactors that impact OGT and OGA catalytic activities and substrate targeting in basal and disease models. Collectively, data arising from these studies should lead to additional biomarkers of disease and highlight points at which O-GlcNAc can be manipulated to improve clinical outcomes.

## Conflict of interest

The authors declare that they have no conflicts of interest with the contents of this article.
